# Energy Metabolism Plays a Critical Role in Stem Cell Maintenance and Differentiation

**DOI:** 10.3390/ijms17020253

**Published:** 2016-02-18

**Authors:** Chenxia Hu, Linxiao Fan, Panpan Cen, Ermei Chen, Zhengyi Jiang, Lanjuan Li

**Affiliations:** Collaborative Innovation Center for Diagnosis and Treatment of Infectious Diseases, State Key Laboratory for Diagnosis and Treatment of Infectious Diseases, School of Medicine, First Affiliated Hospital, Zhejiang University, Hangzhou 310058, China; 11318093@zju.edu.cn (C.H.); 21418049@zju.edu.cn (L.F.); cenpanpan90@163.com (P.C.); cemsmiles@163.com (E.C.); zhengyi_0922@163.com (Z.J.)

**Keywords:** glycolysis, mitochondria, oxidative phosphorylation, stem cells, regenerative medicine

## Abstract

Various stem cells gradually turned to be critical players in tissue engineering and regenerative medicine therapies. Current evidence has demonstrated that in addition to growth factors and the extracellular matrix, multiple metabolic pathways definitively provide important signals for stem cell self-renewal and differentiation. In this review, we mainly focus on a detailed overview of stem cell metabolism *in vitro*. In stem cell metabolic biology, the dynamic balance of each type of stem cell can vary according to the properties of each cell type, and they share some common points. Clearly defining the metabolic flux alterations in stem cells may help to shed light on stemness features and differentiation pathways that control the fate of stem cells.

## 1. Introduction

Stem cells, which can be generated not only from adult tissues but also from embryonic tissues, have gradually turned out to be critical players in regenerative medicine therapies. In addition, terminal somatic cells can be converted from a differentiated state to a pluripotent state similar to embryonic stem cells (ESCs) by overexpressing transcription factors, namely induced pluripotent stem cells (iPSCs) [1]. iPSCs exhibit levels of OCT4 or NANOG that are similar to ESCs so that they display a high level of pluripotency and self-renewal similar to that of ESCs. Both types of cells can indefinitely self-renew and are superior in their primitive stemness, giving rise to virtually any somatic cell type. In contrast, stem cells derived from adult tissue benefit from their easy access and abundant supply, but their potency is limited by their differentiation capacity. 

**Figure 1:**
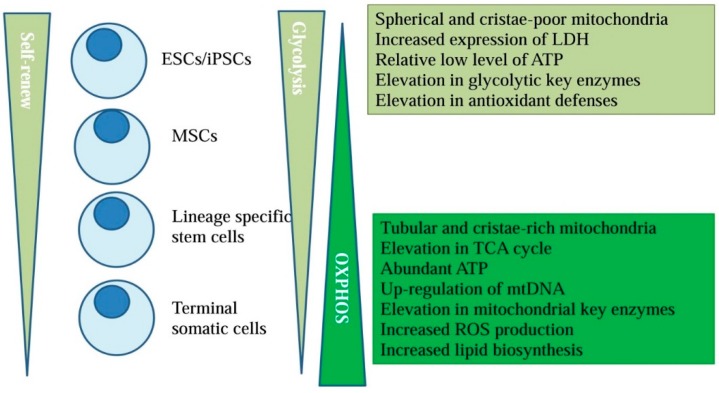
Metabolic pathways may provide important signals to direct the self-renewal and differentiation potency of stem cells.

Current evidence has demonstrated that in addition to growth factors and extracellular matrix cues, various metabolic pathways definitively provide important signals for the self-renewal and differentiation potency of stem cells [2] (Figure 1). The metabolic profile distinguishes the undifferentiated state from the differentiated state of stem cells, with a dynamic mitochondrial morphology and a shift from glycolysis to mitochondrial oxidative phosphorylation (OXPHOS) [3,4,5,6,7]. Glycolysis rapidly fulfills energy requirements by producing pyruvate in the cytosol, which is only accompanied by a net gain of two moles of adenosine triphosphate (ATP) per mole of glucose. However, pyruvate is likely to enter the tricarboxylic acid (TCA) cycle for OXPHOS and generate reducing equivalents to efficiently produce ATP for a significantly higher energy yield than glycolysis [8]. In fact, the mitochondrial metabolites including ATP, intracellular Ca^2+^ homeostasis, and reactive oxygen species (ROS) are crucial for multiple cellular processes. In stem cell metabolic biology, the dynamic balance of each type of stem cell can vary according to the properties of each cell type, and they share some common points. In this review, we mainly discuss how various stem cells metabolize to self-renew and differentiate *in vitro*. Understanding the mitochondrial properties of stem cells may effectively clarify the stemness and differentiation pathways that control stem cells for regenerative medicine. 

## 2. Energy Metabolism and Stem Cell Fate

In mammalian cells, several vital biosynthetic pathways and the generation of ATP are primarily accomplished through the harmonious expression of proteins encoded by nuclear DNA and mitochondrial DNA (mtDNA) [9]. Additionally, the extent, efficacy and coordination of mtDNA processing are pivotal parameters of the mitochondrial status in living cells [10]. ATP demand can be measured with specific analyzers, ATP determination kits or other methods according to different requirements [11,12,13]. Furthermore, defects in the mitochondrial electron transport chain (ETC) are associated with mtDNA damage and will consequently increase ROS production [14]. Uncoupling protein 2 (UCP-2) effectively reduces carbon substrates in OXPHOS by transporting four carbon TCA cycle intermediates out of the mitochondria [15]. The pyruvate dehydrogenase (PDH) complex effectively oxidizes pyruvate to generate acetyl coenzyme a (AcCoA) and CO2 [16]. AcCoA can form citrate by condensing with oxaloacetate and can subsequently be transferred to the cytosol to further metabolize to provide carbon for lipid biosynthesis [16]. The TCA cycle also provides AcCoA for acetylation and lipogenesis, whereas deprivation of nutrients can lead to limited substrates in the cytosol [17]. In addition, mitochondrial glucose oxidation releases ROS, which may lead to accumulated damage and an impaired reconstitution capacity [18]. Further, PGCs are essential for mitochondrial biogenesis and the production of several ROS-detoxifying enzymes after exposure to oxidative stress [19]. The balanced expression of regulatory proteins guarantees genomic stability through the cell cycle activation when stem cells respond to DNA damage or oxidative stress [20].

Highly proliferative stem cells convert pyruvate with lactate dehydrogenase (LDH) to lactate at high rates to meet energy requirements, and consequently, glucose metabolism is kept separate from oxidative metabolism [21,22]. When stem cells divide to proliferate, the older mitochondria are asymmetrically apportioned into one daughter cell, and the younger mitochondria are apportioned to another daughter cell [23]. Then, one cell maintains stem cell characteristics, and the other assumes a more lineage-specific role [24]. After specified lineage differentiation, mtDNA levels and emerging energy requirements are gradually increased in support of mitochondrial biogenesis [25,26]. Indeed, the spherical and cristae-poor mitochondria of undifferentiated stem cells are transformed into tubular and cristae-rich structures to guarantee sufficient ATP for energy metabolism after specific lineage differentiation [26]. Concomitantly, the production of mitochondrial-related key enzymes and mitochondrial ROS are improved, but the expression levels of glycolytic genes and the production of antioxidant defenses are suppressed [26,27,28,29,30]. The addition of saturated metabolites to differentiation media promotes lineage transition, whereas unsaturated fatty acids impair lineage specification by inhibition of the eicosanoid pathway [30]. Furthermore, fatty acid oxidation (FAO) produces one molecule of AcCoA in each cycle and two molecule of AcCoA in the final cycle, with AcCoA-induced oxaloacetate to produce citrate for the generation of NADPH [31]. FAO also acts to maintain enough production of ATP and NADPH to counteract oxidative stress during metabolic stress [32,33]. Along with these, other amino acids and TCA-associated metabolisms were also related to the self-renewal and differentiation of stem cells [34]. 

## 3. Totipotent Stem Cells

Totipotent stem cells (ESCs and iPSCs) refer to stem cells that are highly plastic and can potentially be directed to any cell type. Somatic mitochondrial biology, including organelle morphology and distribution, mtDNA content, expression levels of mitochondrial biogenesis related nuclear factors, intracellular ATP production and lactate generation, reverts to an immature ESC-like state [35]. However, unsaturated fatty acids were expressed at increased levels in ESCs when compared to iPSCs, which indicates that metabolic differences certainly exist in both pluripotent cell types [21]. Although the metabolic characteristics of iPSCs are not exactly the same as those of ESCs, both of them primarily rely on glycolysis to meet energy requirements and in contrast to their somatic counterparts [36]. Compared with mature fibroblasts, rapidly self-renewing ESCs and iPSCs have significantly lower levels of mitochondrial activity, antioxidant enzymes, oxidative proteins, ROS levels and lipid hydroperoxides [25,37,38]. Mitochondria within iPSCs and ESCs are transformed to a mature morphology analogously, and metabolism changes from an anaerobic state to an aerobic state upon differentiation [37]. 

### 3.1. ESCs

ESCs prefer high rates of glycolysis rather than OXPHOS even when they are cultured in conditions with atmospheric oxygen [39]. Although ESCs generally have immature mitochondria, they demonstrate the Warburg effect with high aerobic metabolism in spite of their high lactate generation [39]. In contrast, another study showed that human ESCs generated the majority of ATP through OXPHOS [11]. ESCs can be maintained in various culture media *in vitro*, and pluripotent markers and mitochondrial status are concomitantly altered according to the current microenvironment [40]. Moreover, their enhanced removal capacity helps to ensure low levels of ROS to defend the genomic integrity of ESCs [29]. In one study, although the expression levels of pluripotent markers were indistinguishable in ESCs with different mitochondrial membrane potentials, mouse ESCs with a higher mitochondrial membrane potential exhibited elevated oxygen consumption, improved mammalian target of rapamycin (mTOR) activity and higher secretion of lactate *in vitro* [41]. Regulation of anaplerotic pathways including glutaminolysis and pyruvate carboxylase are always prerequisite for proliferating mouse ESCs to maintain the levels of TCA cycle in energy metabolism [42]. Let-7 serves as an important mediator in energetic metabolism and leads to a down-regulation of the PI3K/AKT/insulin pathway but an up-regulated metabolism of fatty acid [43]. Growth factor erv1-like serves to protect the integrity of structural and functional mitochondria and plays an obligatory pro-survival role in the maintenance of pluripotency in murine ESCs [44], whereas ATAD3B is a negative regulator of the ubiquitous ATAD3A and functions as an adaptor of mitochondrial homeostasis in human ESCs [45].

The activation of glycolysis, accelerated activation of the TCA cycle, activated lipid synthesis, and activation of glutaminolysis are initiated during the early phase of ESC specific differentiation [46]. The abundance of proteins associated with RNA processing and protein folding is higher in undifferentiated human ESCs, whereas the metabolism of proteins associated with redox, vitamin and energy metabolism and ubiquitin dependent proteolysis is more abundant in differentiated cells [47]. Depletion of Ptpmt1 does not influence homeostasis in conditional knockout ESCs, whereas the proliferation and differentiation abilities are likely to decrease through oxygen consumption and enhanced glycolysis concomitantly [48]. Rapamycin acts to inhibit the mTOR activity by decreasing metabolic activity and consequently promotes the mesodermal differentiation of ESCs [49]. Under differentiating conditions, loss of PKC lambda/iota may lead to injury to mitochondrial organization and maturation and a metabolic shift toward glycolysis [50]. Junctophilin2, which physically links the mitochondria to the sarcoplasmic reticulum, is vital for proper mitochondrial function and Ca^2+^ homeostasis in cardiomyogenic differentiation of mouse ESCs [51]. Agonists of peroxisome proliferator-activated receptor a (PPARa), are able to accelerate the cardiomyogenesis of mouse ESCs by increasing ROS production [52]. Ectopic expression of prohibitin 2 in mouse ESCs can result in mitochondrial swelling and inhibit lineage-specific differentiation toward neurons [53]. Moreover, many lipid molecules are expressed differently in undifferentiated ESCs compared to terminal neurons and cardiomyocytes, and consequently, the pluripotency of ESCs can be increased and the expression levels of unsaturated fatty acids can be maintained by inhibiting the eicosanoid signaling pathway [30]. Furthermore, the disruption of the rate-limiting enzyme for FAO may result in decreased ATP production and attenuated resistant ability to nutrient deprivation in fatty acid metabolism in ESCs [54]. 

### 3.2. iPSCs

**Figure 2:**
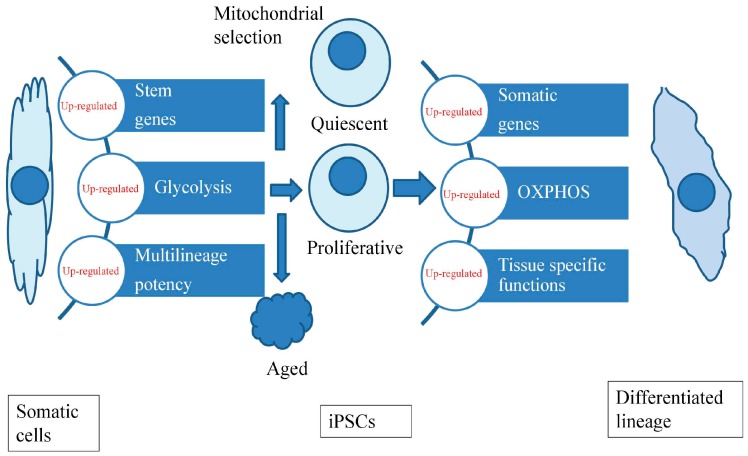
Successful reprogramming is always accompanied by a metabolic shift from a pro-oxidative state to glycolysis, and it will conversely shift after differentiation.

After terminal somatic cells are reprogrammed to a pluripotent state, iPSCs exhibit morphology, gene expression, self-renewal properties and differentiation potential that are almost indistinguishable from those of ESCs. Successful reprogramming is always accompanied by a metabolic shift from an oxidative state to glycolysis, and it will conversely shift after differentiation (Figure 2). Nuclear reprogramming reverts mitochondria to an immature state with an oxidative capacity equivalent to ESCs, whereas greater glycolytic capacity has been found in iPSCs with c-Myc when compared to cells without c-Myc [55]. The estrogen-related receptor (ERR) α and γ, accompanied by their partnered co-factors including peroxisome proliferator-activated receptor-gamma coactivator 1 (PGC-1) α and β are transiently induced and consequently lead to a burst of OXPHOS activity at an early stage of reprogramming [56]. Furthermore, the expressed proteome demonstrates that the protein expression levels of ETC complexes I and IV are reduced during early-stage reprogramming, whereas ETC complexes II, III, and V are momentarily increased in the midterm phase of mouse iPSC generation [57]. mtDNA mutagenesis is considered a critical factor in the reduction of iPSC reprogramming efficiency by increasing mitochondrial H_2_O_2_, and mitochondria-targeted ubiquinone and *N*-acetyl-l-cysteine can efficiently rescue the defects of mtDNA mutagenesis and enhance reprogramming efficacy [58]. In contrast, Prigione *et al.* demonstrated that mtDNA mutations may not necessarily influence the accurate establishment of pluripotency and associated metabolic reprogramming [59]. Aged iPSCs that fail to properly undergo *in vitro* neurogenesis present an increased number of mitochondria per cell [60]. 

By inhibiting glycolysis or promoting oxidative metabolism, the reprogramming process can be impaired, whereas enhancement of glycolysis improves reprogramming efficiency [61]. For example, activation of AMP-activated protein kinase (AMPK) builds a metabolic barrier to reprogramming by shifting away the glycolysis, which fuels the maintenance of stemness [62]. Inhibited expression of dynamin-related protein 1 (DRP1) sustains the fused mitochondrial network and inhibits iPSC reprogramming [63], whereas shRNA knockdown of DRP1 does not impair iPSC reprogramming but only leads to mitochondrial fusion [64]. REX1, which increases the phosphorylation and activation of DRP1, fission of the mitochondrial network and glycolytic metabolism in iPSCs, is required to maintain self-renewal [65]. By down-regulating expression of the mitochondrial inner membrane protein, reprogramming efficiency can be significantly reduced [66]. Additionally, an inhibitor of pyruvate dehydrogenase kinase (PDK) activity named dichloroacetate decreases pluripotent iPSC generation by increasing pyruvate transport into the mitochondria and TCA metabolism [67]. Mitochondrial inhibition effectively converts the refractory intermediates to pluripotent states without supernumerary genetic or epigenetic modifications [67,68]. Furthermore, the addition of antioxidants into the culture medium of human iPSCs enhances genomic stability, repairing DNA damage and maintaining low ROS [69]. 

According to two-dimensional differential gel electrophoresis, half of the identified proteins, which are differentially expressed in iPSCs and the differentiated cells, are localized in the mitochondria and participate in metabolic kinetics and pluripotent regulation [70]. Expression levels of PDH phosphorylation and 3-phosphoinositide dependent protein kinase-1 (PDK1), which are likely to reduce oxidation of glucose carbon in the TCA cycle, are significantly higher in human iPSCs compared with terminally differentiated fibroblasts [36]. iPSCs proliferate slower after differentiation, accompanied by a progressively fused mitochondrial network, decreased glycolysis, increased respiratory capacity and improved mitochondrial oxidation [71]. Inhibition of the mitochondrial permeability transition pore by cyclosporin A demonstrates an increased expression of mitochondria-related genes, mitochondrial calcium, ATP level, mitochondrial membrane potential, and oxygen consumption rate (OCR), which consequently leads to promotion of cardiomyogenic differentiation of iPSCs [72]. When iPSCss from familial Parkinson’s disease patients were differentiated to neural cells, they demonstrated cellular vulnerability related to mitochondrial dysfunction, but the defects could be recovered by coenzyme Q(10), rapamycin or the LRRK2 kinase inhibitor GW5074 [73].

## 4. Mesenchymal Stem Cells (MSCs)

Adult somatic stem cells are fibroblast-like and non-hematopoietic, and they can be isolated from adult somatic tissues including bone marrow, adipose, placenta, umbilical cord, umbilical cord blood and other resources [74]. They have emerged as a useful tool without ethical issues in regenerative medicine; they have lower tumorigenicity compared with ESCs and iPSCs, and their potency and organ availability are higher than that of lineage-specific stem cells [74]. 

In undifferentiated MSCs, mitochondrial activities are maintained at a low level, but glycolytic activities are consistently maintained at a high level for a majority of glycolytic enzymes and lactate production [75]. In detail, glycolysis contributes to greater than 97% of ATP production, whereas OXPHOS contributes less than 3% of ATP production in the energy metabolism of undifferentiated bone marrow MSCs [76]. Consistent with the metabolic signature, the reduction of saturated FAO can reduce human bone marrow MSC proliferation and cause cell death to a certain extent [76]. During hepatocyte maturation of MSCs, the expression levels of major polyunsaturated fatty acids decreased but the expression levels of saturated fatty acids increased; however, these alterations did not depend on ROS production and lipid peroxidation in differentiating cells [77]. Linoleic and oleic acids are able to inhibit MSC proliferation and altered the secretion of interleukin-6, VEGF and nitric oxide [78]. After MSCs were cultured long-term *in vitro*, the down-regulated levels of genes associated with cytoskeleton, mitochondria function, focal adhesion and differentiation simultaneously resulted in alteration of mitochondrial morphology, decreased levels of antioxidants and increased levels of ROS [79]. 

During the early stages of MSC differentiation, the new cell fate is redirected by down-regulating the pluripotent specific genes, up-regulating the terminal-specific genes and switching the subsets of metabolic enzymes [80]. The mtDNA copy number, content of respiratory enzymes, intracellular ATP and OCR increase, but the levels of intracellular ROS dramatically decrease in osteogenic MSCs, and exogenetic addition of mitochondrial inhibitors can delay the osteogenesis of MSCs [81]. Fluorescence lifetime imaging of NADH has been positively correlated with OCR and ATP production during transition of glycolysis to OXPHOS [82]; JC-1 fluorescence has also been found to be strongly correlated with the osteogenic differentiation ability of MSCs [83]. Hypoxia inducible factor (HIF)-1α participates in regulating the metabolic fate and multipotency of human MSCs, and after osteogenic differentiation, the expression of HIF-1α is reduced and leads to decreased glycolytic metabolism and increased oxidative metabolism [84]. The mitochondrial ROS production released by the ETC was able to initiate or enhance adipogenic differentiation in MSCs [38]. The antioxidants catalase and superoxide dismutase (SOD) increase at day seven of adipogenic differentiation, and furthermore, adipogenic differentiation of human MSCs has been demonstrated to show a shift toward higher OCR [27]. The addition of mitochondrial-targeted antioxidants MitoCP/MitoCTPO or knockdown of the Rieske Fe-S protein of complex III reduce ROS production and impair adipocyte lineage specification [38]. Overexpression of miR-27a or miR-27b leads to inhibited prohibitin expression and prohibitive adipocyte differentiation by impairing mitochondrial biogenesis and accumulating ROS production [85]. 

## 5. Lineage-Specific Stem Cells

### 5.1. Hematopoietic Stem Cell (HSCs)

The mammalian HSC system consists of quiescent long-term (LT)-HSCs, short-term (ST)-HSCs, multipotent progenitors and various lineage-restricted progeny [86]. When HSCs in whole bone marrow are less differentiated, they exhibit fewer mitochondria and higher glycolytic capacity [5,87,88], and the levels of antioxidant enzymes including superoxide dismutase, catalase and glutathione peroxidase are higher in circulating progenitor cells than in LT-HSCs [89]. An enhanced glycolytic status promotes LT-HSC cell cycle quiescence *in vitro* and *in vivo* [7]; mitochondria and levels of ROS cooperate to balance self-renewal and cell division in cycling HSCs [90]. Moreover, 12/15-lipoxygenase-dependent fatty acid metabolism maintains the quiescence of long-term HSCs, and the defect in HSCs is related with reductive production of bioactive lipid mediators and ROS and with a decreased Wnt signaling [91]. Peroxisome proliferator-activated receptors regulate critical enzymes in FAO. The deletion of PPARδ leads to poor HSC self-renewal, whereas the pharmacologic activation of PPARδ promotes self-renewal and asymmetric division [92]. Depletion of Ppard or Pml, accompanied with inhibited mitochondrial FAO, leads to symmetrically committed HSCs *in vitro* and *in vivo* [93]. The deletion of the cytoskeleton-modulating protein profilin 1 (pfn1) in HSCs leads to a switch from glycolysis to OXPHOS with increased ROS levels and then results in loss of quiescence and apoptosis of HSCs *in vivo* [94]. Aberrant ROS generation abrogates stem cell properties including quiescence, self-renewal, and survival as well as the multi-lineage capacity of HSCs [95]. Indeed, lead acetate perturbs the hematopoietic balance of adult HSCs by increasing intracellular ROS generation and resulting in cellular mitochondrial defects [96]. FoxOs mitigates the ROS levels of hematopoietic progenitors, which results in cell cycle arrest, cell apoptosis and oxidative stress resistance in HSCs [97]. Deletion of SOD2 or suppression of the ND75 subunit of complex I in hematopoietic progenitors by increasing ROS production over general levels may induce progenitors from a premature status to a mature status. Furthermore, overexpression of GTPx-1 or catalase damages lineage-specific differentiation by reducing ROS production [98]. As HSCs in bone marrow age, there is an increase in intracellular superoxide anions, hydrogen peroxide, nitric oxide, and peroxynitrite/hydroxyl compared with young cells [99].

### 5.2. Neural Stem Cells (NSCs)

Endogenous NSCs, which exist in specific niches of the brain, restore neurons for the maintenance of normal conditions and functions [100]. Even under physiologically normoxic O_2_ conditions, the levels of mitochondrial oxidative metabolism, glycolysis and ROS in NSCs have been observed to be similar to those exposed to 20% O_2_, but cell populations in normoxic O_2_ conditions possess better resistance to *in vitro* inflammatory injury [101]. NSCs have been demonstrated to be dependent on fatty acid synthase-mediated lipogenesis for the proliferation and neurogenetic differentiation [102]. Inhibition of the rate-limiting enzyme of oleic acid synthesis rescues proliferative impairments of adult neurogenic niches in Alzheimer's disease mice [103]. Polyunsaturated fatty acid promotes NSCs to express FAO related enzymes and continuously increase the oxygen consumption; after treatment with etomoxir, an inhibitor of FAO, the oxygen consumption and the proliferation of NSCs decreases but the cellular survival is not altered [104]. Increased expression of fatty acid synthase redirects fatty acid metabolism for the support of the anabolic requirements of proliferating stem cells. In contrast, inhibition or deletion of fatty acid synthase reduces proliferation of NSCs [102].

In accordance with the dynamic regularity of other stem cells, the mitochondrial mass, mtDNA copy number and respiration capacity are robustly improved after the differentiation of NSCs [105]. Ca^2+^-mediated ROS metabolic cues regulate the differentiation efficiency by regulating the Wnt/β-catenin signaling pathway [106], and the inhibition of the mitochondrial permeability transition pore, which serves as a signaling regulator, suppressing neuronal differentiation [107]. The increased respiration activity constrains mtDNA in NSCs vulnerable to oxidative damage, and defects in mitochondrial 8-oxoguanine DNA glycosylase function leads to the accumulation of mtDNA damage during differentiation [105]. Although there is no alteration of mitochondrial mass in NSCs, mitochondrial biogenesis increases after initiation of human NSC differentiation into motor neurons [108]. Hepatocyte growth factor and metallothionein 2 are always expressed at lower levels in neural stem cells without functional transcripts of Prdm16 [109,110,111]. However, genetic mitochondrial damage is not likely to alter the generation, maintenance or multipotency of glia-like central NSCs [112]. Intriguingly, oligodendrocyte lineage cells contribute considerably to the metabolic activity of the central nervous system at late differentiation stages [113]. 

## 6. Hypoxia, Energy Metabolism and Stem Cell Fate

Niches provide essential factors for the maintenance of self-renewal and prevention of differentiation in stem cells, and these niches are where oxygen concentration is extremely low and stem cells are rigorously regulated [114,115]. Because oxygen serves as the final electron acceptor of OXPHOS, aerobic metabolism is fundamental in mammalian cells under normoxic environments [116]. For most cell types, hypoxia serves as a modulator of cell proliferation and has been found to decrease the levels of respiratory enzymes and OCR but increase the production of glycolytic enzymes and lactate, which eventually forces the cells to rely more on glycolysis [117]. When the availability of molecular oxygen is limited under hypoxic conditions, the activity of ETC is decreased, and energetic needs are likely to shift from OXPHOS to glycolysis, and consequently, stem cells enhance their self-renewal ability and maintenance of pluripotent capacity *in vitro* [118]. Although hypoxia enhances the proliferative ability of stem cells and reduces their differentiation potency [40,41], opposing observations have demonstrated that oxygen has minimal effects on undifferentiated cell growth and phenotype but becomes influential under differentiating conditions [119]. In addition, the levels of pluripotency and terminal markers, accompanied by proliferation ability have been shown to be unaltered at 5% and 20% oxygen concentrations, with the apoptosis rate elevated under 5% oxygen conditions [120]. ESCs increase anaerobic metabolism and survive oxygen starvation with negligible cell death, but the total ATP production remains almost constant under hypoxic conditions [119]. MSCs have been demonstrated to be maintained in an undifferentiated state through the suppression of mitochondrial activity in hypoxia [121], and they retained the ability to be differentiated into chondrocytes, adipocytes and cardiomyocytes under hypoxic or ischemic conditions [122]. Under normal metabolic conditions, ROS are produced in small quantities, but they can be significantly increased after acute inhibition of the ETC or exposure to hypoxic environment [123].

Hypoxia directly decreases ETC activity, not only by reducing oxygen concentration but also by activating the expression of HIFs [37,38,39]. The transcription factors of HIFs reduce the expression levels of mitochondrial enzymes and further up-regulate glycolytic enzymes and glucose transporters [38,39]. HIFα proteins (HIF-1α, HIF-2α, and HIF-3α) demonstrate tissue-specific expression levels and various functions [124] and are closely correlated with regulation of pluripotency factors [125]. However, the role of the HIF family in the maintenance of pluripotent ability has emerged as a controversial issue [126]. HIF-1 has been proven to inhibit the differentiation of ESCs [127], and HIF-2 regulates the proliferation rather than self-renewal in stem cells [125]. When MSCs are cultured under hypoxic conditions, the expression of HIF-1α and energy metabolism-associated genes are increased [128,129]. 

## 7. Conclusions

Self-renewal and differentiation abilities vary in stem cells, and the regulation of metabolic pathways has been demonstrated to take part in regulating stem cell fates. We have here highlighted the general norms of stem cell metabolism, but there are numerous important questions that remain to be answered in stem cell biology: (1) How can we improve the function of stem cells by regulating intracellular metabolism? (2) How can we improve reprogramming efficacy with metabolic cues for direction of terminal somatic cells to high pluripotent cells? (3) Can we gain the comprehensive understanding of how metabolic signaling molecules cooperate to regulate the stem cell lineage-specific differentiation that is necessary for tissue engineering? (4) Will we discover if the energy metabolism of tissue-derived stem cells including MSCs, HSCs and NSCs is similar to that of totipotent stem cells? (5) What is known about the metabolic characteristics of adult somatic stem cells derived from other resources? Heretofore, researches have mainly focused on bone marrow-derived MSCs; (6) It is necessary to clarify the subnets of metabolic biology in hypoxic environments for the regulation of stem cell development. Because of its critical role in regulating the self-renewal and differentiation process of stem cells, metabolism will facilitate the optimization of *in vitro* maintenance and differentiation protocols by adjusting biochemical properties for regenerative medicine.
